# The Economic Value of the Penny

**Published:** 1918-09-21

**Authors:** 


### The Economic Value of the Penny.

At the Leicester and County Saturday Hospital Society's half-yearly meeting, held at the Royal Infirmary, with the President, Mr. J. Gibson Clarke, in the chair, the Committee's report referred to the serious economic fact that the purchasing power of the penny had considerably decreased, with the consequent result that the advantages the Society offered to the penny and halfpenny contributor were very much in the members' favour. The Committee, therefore, might reluctantly have to ask the members to double their weekly contributions. The work showed no diminution. The total expenditure was £3,794 as compared with £3,109. The Committee had, in lieu of a collection from the members, responded to an appeal from the local Prisoners of War Fund by making a grant of £150. This action was dictated by the possibility that some members of the Society might be prisoners of war.

The President, in moving the adoption of the report, hoped it would not be necessary to increase the weekly contributions. An opportunity had arisen to purchase the Society's offices, and in view of the manifest importance of retaining premises which were convenient, central, and well known, the freehold had been acquired at a satisfactory figure. Satisfaction was expressed that the name of Miss L. M. Havers, matron of the Desford Auxiliary Hospital, had been brought to the notice of the Secretary of State for War for meritorious service. Her health had unfortunately failed, and for this and domestic reasons she had resigned. Miss Havers had been matron since the Home was first opened some thirteen years since. Miss Morley, the acting matron, had been appointed matron until January. The report was adopted.

Mr. Gimson then handed to Mr. T. Fielding Johnson, jun., chairman of the board of the infirmary, a cheque for £728 14s. 8d., a first payment on account of the members' collection for the "Sir Edward Wood" Memorial Fund. The members' tribute to their first President and founder was received with gratitude by Mr. Fielding Johnson. The proposed memorial to Sir Edward Wood was the rebuilding of the south-west wing, erected in 1861, and not now up to modern requirements. Alluding to the pressing need for an Orthopædic Department in Leicester for the treatment of discharged soldiers, he stated he was hopeful that a favourable decision would be given by the Ministry. The temporary out-patients' department was treating increasing numbers each week, and had become quite inadequate. There was no orthopædic hospital in the North Midland area, and only in the last few days discharged soldiers who had been receiving treatment at a smaller local hospital had been referred to the infirmary for treatment, by the direction of the Ministry.

